# ‘Potentially curative therapies’ for hepatocellular carcinoma: how many patients can actually be cured?

**DOI:** 10.1038/s41416-023-02188-z

**Published:** 2023-02-17

**Authors:** Alessandro Cucchetti, Omar Elshaarawy, Guohong Han, Charing C. N. Chong, Carla Serra, Joanne Marie O’Rourke, Richard Crew, Cristina Felicani, Giorgio Ercolani, Tahir Shah, Arndt Vogel, Paul B. S. Lai, Philip J. Johnson

**Affiliations:** 1 Department of Medical and Surgical Sciences, Alma Mater Studiorum, University of Bologna, Bologna, Italy; 2 Morgagni—Pierantoni Hospital, Forlì, Italy; 3 National Liver Institute, Shebeen El-Kom, Egypt; 4 Department of Liver Disease and Digestive Interventional Radiology, Xijing Hospital, Fourth Military Medical University, Xi’an, China; 5 Division of Hepatobiliary and Pancreatic Surgery, Department of Surgery, Prince of Wales Hospital, The Chinese University of Hong Kong, Ma Liu Shui, Hong Kong SAR; 6 IRCCS Azienda Ospedaliero-Universitaria di Bologna. S. Orsola—Malpighi Hospital, University of Bologna, Bologna, Italy; 7 The Liver Unit, University Hospitals Birmingham NHS Foundation Trust, Birmingham, UK; 8 Technology, Infrastructure & Environment Directorate, Faculty of Health & Life Sciences, University of Liverpool, Liverpool, UK; 9 Clinic for Gastroenterology, Hepatology and Endocrinology, Hannover Medical School, Hannover, Germany; 10 Department of Molecular and Clinical Cancer Medicine, University of Liverpool, Liverpool, UK

**Keywords:** Hepatocellular carcinoma, Liver cancer

## Abstract

**Background:**

Treatment of hepatocellular carcinoma (HCC) is predicated on early diagnosis such that ‘curative therapies’ can be successfully applied. The term ‘curative’ is, however, poorly quantitated. We aimed to complement our previous work by developing a statistical model to predict cure after ablation and to use this analysis to compare the true curative potential of the various ‘curative’ therapies.

**Methods:**

We accessed data from 1571 HCC patients treated in 5 centres receiving radiofrequency (RFA) or microwave (MWA) ablation and used flexible parametric modelling to determine the curative fraction. The results of this analysis were then combined with our previous estimations to provide a simple calculator applicable to all patients undergoing potentially curative therapies.

**Results:**

The cure fraction was 18.3% rising to about 40% in patients with good liver function and very small tumours.

**Conclusion:**

Cure for HCC treated with ablation occurs in the order of 20% to 30%, similar to that achievable by resection but much inferior to transplantation where the analogous figure is >70%. We provide a ‘calculator’ that permits clinicians to estimate the chance of cure for any individual patient, based on readily available clinical features.

## Introduction

Treatment of hepatocellular carcinoma (HCC) is predicated on early diagnosis such that the ‘potentially curative therapies’ (PCTs) of hepatic resection (HR), liver transplantation (LT) or ablation can be successfully applied [1, 2]. However, the term ‘potentially curative’ is vague and poorly quantified. From a technical point of view, ‘cure’ takes place when a population treated from a specific disease obtains the same life-expectancy as a reference population that has never had the disease [3, 4]. Recently, statistical models have been provided that permit the quantification of the actual chance of cure for two of the three potentially curative treatments [LT [3] and HR [4]]. From these studies the superiority of LT is clear with a chance of cure in the region of 70%. However, in a disease such as HCC, LT cannot be considered a practical solution to the vast global problem of HCC because of costs, limited infrastructure and the chronic shortage of donor organs. Long term control of chronic liver disease by immunisation (in the case of HBV), anti-viral therapies (HBV and HCV) and surveillance, together with other public health measures, are likely to have the major impact.

On the other hand, resection offers only a 25% chance of cure, the difference reflecting both the emergence of initially undetected intrahepatic metastases that lead ultimately to disease recurrence and continuing morbidity from liver dysfunction [4]. Nonetheless, HR is regarded as first line treatment despite the risk of postoperative complications and recurrence. As an alternative, or complementary to, local ablative therapies such as radiofrequency ablation (RFA), microwave ablation (MWA) and, previously, percutaneous ethanol injection (PEI) have become increasingly applied. The low incidence of complications and the high tumour control rates (particularly in patients with small tumours (<3 cm) [5] make such ablative approaches more cost-effective than resection [6]. At this tumour size, survival analysis in prospective controlled trials appears similar to that achievable by resection [7]. Liver function represents the second factor impacting the outcome of PCTs, so that the higher the degree of liver dysfunction, the lower the probability of receiving HR, shifting the therapeutic choice to ablation therapies. In this regard, treatment selection is conventionally based upon the Child-Pugh score (CPS) but a refinement of this method, the Albumin-Bilirubin (ALBI) score [8], appears at least as good in terms of prognostic ability but provides more objective and granular data that can be readily integrated into a statistically based scoring system.

Herein we first undertake a detailed assessment of the curative potential of the third PCT treatment, namely ablation, through the application of a ‘statistical cure’ model. Then we combine all three models into a single calculator such that the clinicians can determine the outcome, in terms of likelihood of cure, for any of the three PCT treatments in an individual patient.

## Patients and methods

The present study population was derived from an international retrospective cohort including a total of 1655 patients treated with ablation, either RFA or MWA, for HCC from 5 centres in different countries both Eastern and Western between February 2004 and November 2018 with follow-up to September 2019. All centres had extensive experience of managing HCC. Informed consent was obtained by all patients, and their data fulfilled ethical requirements according to local practice and the present study fulfilled the Regulation (EU) 2016/679 of the European Parliament and of the Council of 27 April 2016 regarding the processing of personal data.

Ideal candidates for ablation were identified on the basis of international guidelines in place during the study period. The final choice was however personalised according to the perceived probability of survival benefit within the concept of ‘stage migration’ [1]. The present analysis, however, included only patients undergoing ablation with potential curative intent as first treatment of HCC. Consequently, patients with macro-vascular invasion, extra-hepatic disease and Child–Pugh>B8 (*n* = 84) were excluded. Clinical and tumoral data identifying ideal candidates for ablation were then considered when modelling the probability of cure. All patients had complete chronological, clinical, survival and tumour-related data, leading to a final dataset of 1571 patients from Hong Kong (*n* = 337), mainland China (*n* = 549), Italy (*n* = 306), United Kingdom (*n* = 287) and Germany (*n* = 92). The list of retrieved variables is reported in the Table 1. The study population was further divided into three eras as a proxy for progressive technical improvement in ablation techniques, as well as the introduction of modern anti-hepatitis B and C virus therapies [4].

**Table 1:** Characteristics and survivals of the study’ patients ablated for naive HCC.

Variables	*n* = 1571
Age [years; median (IQR)]	62 (54–70)
<60 years	670 (42.6%)
60–70 years	538 (34.2%)
71–80 years	305 (19.4%)
>80 years	58 (3.7%)
Male gender	1197 (76.2%)
Eastern patients	886 (56.4%)
Year of ablation	
2004–2008	166 (10.6%)
2009–2013	524 (33.3%)
2014–2018	881 (56.1%)
Etiology	
Hepatitis B	774 (49.5%)
Hepatitis C	359 (22.9%)
Alcohol	235 (15.0%)
Other	253 (16.1%)
Total bilirubin [μmol/L; median (IQR)]	16.0 (11.0–23.8)
Serum albumin [g/L; median (IQR)]	40 (36–44)
ALBI grade	
1	592 (37.7%)
2	866 (55.2%)
3	111 (7.1%)
MWA	217 (13.8%)
Largest tumour size [cm; median (IQR)]	2.5 (1.9–3.4)
Tumour number	
Single	1176 (74.9%)
2 or 3 nodules	340 (21.6%)
4+ nodules	55 (3.5%)
BCLC very-early stage	317 (20.2%)
Milan IN	1358 (86.4%)
Disease-free survival [years; median (IQR)]	1.3 (0.5–3.6)
1 year (95% C.I.)	58.5% (55.9–60.9)
3 years (95% C.I.)	30.4% (27.9–32.9)
5 years (95% C.I.)	19.1% (16.4–21.4)
10 years (95% C.I.)	8.8% (6.1–12.3)
Overall survival [years; median (IQR)]	5.0 (2.2–9.6)
1 year (95% C.I.)	90.2% (88.5–91.6)
3 years (95% C.I.)	67.5% (64.8–70.2)
5 years (95% C.I.)	49.9% (46.6–53.2)
10 years (95% C.I.)	20.8% (16.0–26.1)

### Primary and secondary aims

The primary aim of the present study was to estimate cure probabilities after RFA/MWA. To accomplish this aim, the disease-free survival (DFS) was applied as the reference survival measure for the cure model, since it would be inappropriate to define as ‘*cured*’, a patient who, even if alive, has tumour recurrence, as it would be if considering overall survival (OS) [4]. Hepatic resection and liver transplantation were considered censoring events. For each country, the reference population considered was the general population. Mortality rates by age, sex, race and year were extracted from national life tables as published for individual countries in the World Health Organization (WHO) database.

We also estimated the years of life lost (YLL) after treatment of HCC through RFA/MWA. The loss in expectation of life metric was calculated by the difference between the mean survival for people without cancer for a given set of characteristics (age, calendar year, sex and race) and the estimated mean survival for patients treated for HCC having the same characteristics.

### Follow-up and definition of recurrence

After ablation, patients were followed according to the local practice of each participating centre. Post-procedural monitoring involved AFP determination, ultrasound and contrast-enhanced computed tomography (CT). In particular, within the first year after ablation CT scan was carried out to verify the complete response of the tumour and to diagnose early recurrence. Subsequently, CT scanning was used less, preference being given to ultrasound as the surveillance method for late recurrences. Computed tomography or magnetic resonance were always applied for the definitive confirmation of tumour relapse, defining the temporal end-point of this main outcome measure. Recurrence of HCC after ablation was defined as the appearance of a new nodule/s, different to the target lesion/s, during follow-up. The presence of incomplete ablation was re-treated with either RFA, ethanol injection or TACE. When, at the end of the therapeutic sequence, there was still viable tumour tissue, patients were deemed to have tumour relapse. Both events counted as tumour recurrences. Disease-free survival counted both recurrence and death as events.

### Statistical analysis

Categorical variables are reported as number of cases and percentages and compared using Fisher’s exact test as necessary. Continuous variables are reported as medians and interquartile ranges (IQR: 25th and 75th percentiles), and differences between the subgroups were compared with the Mann–Whitney test. Median follow-up was assessed by the application of the reverse Kaplan–Meier estimator.

Before applying the cure model, the DFS curve was explored to confirm plausibility of cure. This requisite is fulfilled when the survival curve flattens on the *y*-axis with the passing of time [9]. The DFS curve showed a plateau during follow-up confirming that a cure model was applicable. The cure model used here was a flexible parametric survival model, which predicts survival of the present cohort relative to that of the reference population(s). Simple and multivariable cure models were performed, using variables that had a non-negligible effect (*p* < 0.10) as the simple approach. The cure model also allowed for estimation of years-of-life lost (YLL) [10]. These estimations were subsequently re-fitted with a generalised linear model (GLM) to produce an approximation useful for personalised calculation. All the analyses were conducted using Stata software (StataCorp. 2017. Stata Statistical Software: Release 15. College Station, TX: StataCorp LLC.).

## Results

Characteristics of the study population formed by the 1571 patients are detailed in Table 1. Most patients were treated with RFA (*n* = 1354; 86.2%). Only a minority received MWA (*n* = 217; 13.8%) although this percentage showed an increase over time (2004–2008: 0/166 (0%); 2009–2013: 62/524 (11.8%); 2014–2018: 155/88 (17.6%); fisher exact test, *p* = 0.001). Practically all hepatitis B patients were ablated after the introduction of modern anti-viral therapy (*n* = 764/777; 98.3%), as well as a large majority of hepatitis C patients (*n* = 303/359; 84.4%).

The median follow-up after ablation was 4.7 years (IQR: 2.4–7.8) during which period 925 patients had recurrence (58.9%) and 629 died (40.0%; of these, 459 had recurrence [73.0%]). The median DFS was 1.3 years (IQR: 0.5–3.6) and the median OS was 5.0 years (IQR: 2.2–9.6).

The overall cure fraction was 18.3% (95% C.I.: 15.6–21.1) representing the estimated proportion of ablated patients who will have a survival without tumour relapse equal to the survival of the general population (i.e., achieved ‘statistical cure’). However, it takes 10 years before HCC cure can be claimed with 90% of certainty. In the whole cohort, patients undergoing ablation lost a median of 17.2 years of life (IQR: 11.9–22.9).

### Determinants of cure after ablation

Cure probabilities by clinical and demographic data are detailed in Table 2. Age, alcohol and MWA did not modify cure achievement (*p* > 0.5). On the other hand, females showed higher probabilities of being cured (*p* = 0.014), as well as patients who were seronegative for hepatitis C (HCV-Ab) (*p* = 0.033) and hepatitis B (*p* = 0.043). Cure probabilities decreased as the ALBI grade and tumour burden increased (*p* = 0.001, in both cases). Ablation of very-early HCCs led to a cure fraction of 30.9% (95% C.I.: 24.9–37.1).

**Table 2:** Cure proportions and years of life lost (YLLs) resulting from flexible parametric cure model.

Variables	Proportion cured (95% C.I.)	*p*	Median age (IQR)	Median YLL (IQR)	*p*
Age					
<60 years	19.0% (15.7, 22.7)	Ref.	52 (47, 56)	23.7 (21.1, 27.7)	Ref.
60–70 years	17.4% (13.9, 21.3)	0.470	65 (62, 68)	15.4 (13.1, 17.2)	0.001
71–80 years	17.2% (12.6, 22.5)	0.510	74 (72, 77)	9.4 (7.8, 10.6)	0.001
>80 years	23.4% (10.6, 39.3)	0.555	83 (81, 85)	4.4 (3.5, 4.9)	0.001
Gender					
Male	16.9% (14.2, 19.8)	Ref.	61 (53, 69)	17.4 (12.1, 22.7)	Ref.
Female	23.0% (18.2, 28.1)	0.014	65 (56, 73)	16.6 (11.3, 22.7)	0.144
Year of diagnosis					
2004–2008	14.7% (10.2, 20.2)	Ref.	62 (53, 69)	18.7 (13.6, 25.3)	Ref.
2009–2013	17.5% (14.3, 21.1)	0.346	61 (52, 69)	18.0 (12.5, 23.9)	0.206
2014–2018	20.5% (16.8, 24.4)	0.056	62 (54, 70)	16.1 (11.4, 21.7)	0.001
Hepatitis B					
Negative	20.1% (16.8, 23.6)	Ref.	67 (59, 73)	14.7 (10.2, 19.9)	Ref.
Positive	16.1% (13.0, 19.5)	0.043	57 (50, 65)	20.0 (15.0, 25.1)	0.001
Hepatitis C					
Negative	19.3% (16.4, 22.3)	Ref.	61 (52, 69)	17.2 (12.3, 23.5)	Ref.
Positive	14.4% (10.6, 18.8)	0.033	64 (57, 74)	16.6 (10.2, 22.4)	0.001
Alcohol					
Negative	18.6% (15.9, 21.6)	Ref.	61 (53, 70)	17.6 (12.0, 23.4)	Ref.
Positive	15.8% (10.9, 21.6)	0.323	65 (58, 69)	15.5 (12.1, 20.4)	0.002
ALBI grade					
1	23.8% (19.8, 28.0)	Ref.	60 (51, 69)	16.5 (11.7, 22.6)	Ref.
2	14.8% (12.0, 18.0)	0.001	63 (56, 71)	17.0 (12.2, 22.9)	0.573
3	12.0% (6.8, 18.9)	0.003	61 (53, 69)	20.0 (13.5, 25.4)	0.002
Ablation technique					
RFA	18.5% (15.8, 21.5)	Ref.	61 (53, 69)	17.6 (12.2, 23.4)	Ref.
MWA	16.7% (11.7, 22.3)	0.498	67 (59, 71)	15.7 (11.5, 20.2)	0.001
Largest tumour size					
<2 cm	26.9% (21.7, 32.3)	Ref.	62 (53, 70)	15.1 (10.6, 20.9)	Ref.
2–3 cm	19.2% (15.8, 23.0)	0.007	61 (54, 70)	17.4 (12.0, 22.5)	0.006
3.1–5 cm	12.7% (9.5, 16.4)	0.001	63 (55, 70)	17.5 (12.5, 23.8)	0.001
>5 cm	4.6% (1.9, 9.4)	0.001	56 (48, 67)	23.7 (16.2, 31.2)	0.001
Tumour number					
Single	20.8% (17.8, 24.0)	Ref.	62 (53, 70)	16.7 (11.8, 22.4)	Ref.
2 or 3 nodules	11.5% (8.1, 15.5)	0.001	62 (55, 70)	18.3 (12.2, 24.2)	0.037
4+ nodules	2.5% (0.7, 6.3)	0.001	63 (57, 69)	19.5 (14.6, 24.6)	0.012
Very-early stage					
Within	30.9% (24.9, 37.1)	Ref.	61 (53, 70)	14.5 (9.9, 20.4)	Ref.
Beyond	15.4% (12.8, 18.2)	0.001	62 (54, 70)	17.7 (12.3, 23.7)	0.001
Milan criteria					
Within	20.7% (17.7, 23.8)	Ref.	62 (54, 70)	16.5 (11.5, 22.2)	Ref.
Beyond	5.2% (3.0, 8.1)	0.001	60 (52, 68)	20.8 (14.5, 26.9)	0.001

Variables affecting cure proportion entered into the multivariable flexible parametric model. Variables affecting YLLs were used through the generalised linear model to produce approximated YLLs values. Very-early stage and Milan criteria were not entered in the models because their components (size and number) were already retained.

### Years of life lost

To fully understand the loss of life-expectancy, cure probabilities need to be considered together with the age at diagnosis (Table 2). Consequently, older patients showed lower YLLs than younger ones (*p* = 0.001). Male and females had similar YLLs (*p* = 0.144) because the higher cure proportion of females was counteracted by their older age at diagnosis. Focusing attention on clinical situations with the lowest YLL, it was observed that patients aged >70 years had a median YLL of 8.8 years (IQR: 7.0–10.2), those treated for a very-early stage had a median of 14.5 YLL (IQR: 9.9–20.4) and negative HBV patients had a median of 14.7 YLL (IQR: 10.2–19.9).

### Comprehensive evaluation of cure probabilities

**Fig. 1:**
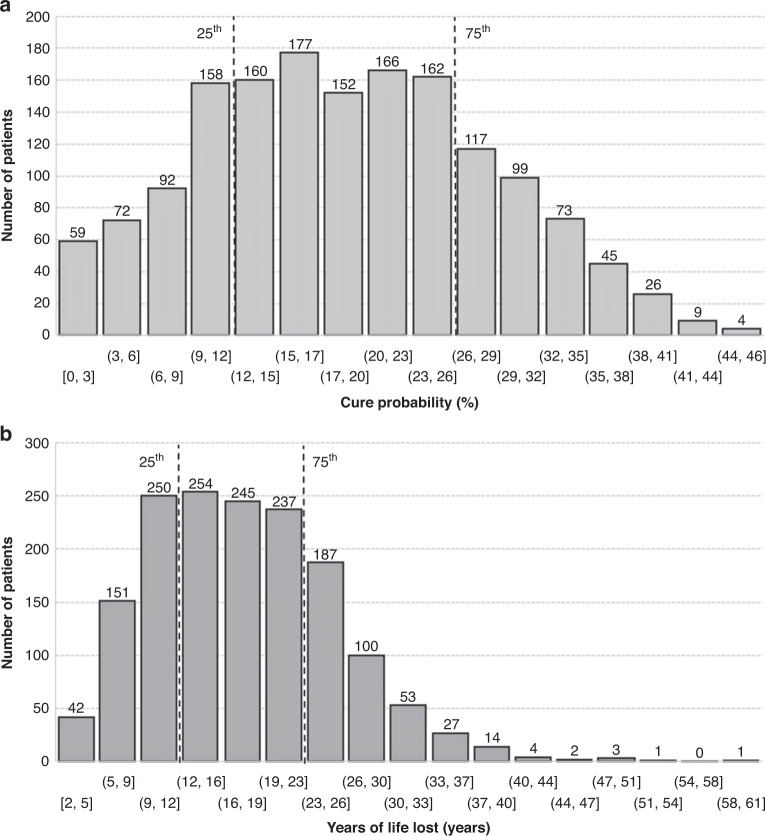
Probability of cure and years of life lost. **a** Estimated cure probabilities and **b** Estimates of YLLs derived from the flexible parametric cure model. The 25th and 75th confidence intervals are shown by dotted vertical lines.

Multivariable flexible parametric survival regression retained gender, year of diagnosis, hepatitis C, ALBI grade, tumour size and number as independent predictors of cure (Table 3). Estimated cure probabilities are depicted in Fig. 1a. The 25th percentile corresponded to a cure fraction of 11.9% and the 75th percentile to a value of 26.0%.

**Table 3:** Results from multivariable flexible parametric survival model on cure probability and from generalised linear model on YLLs.

Variables	Cure-probability model		YLLs approximation	
	Coef. (95% C.I.)	*p*	Coef. (95% C.I.)	*p*
Age (per year)	**-**	**-**	–0.653 (–0.660, –0.646)	0.001
Gender male	0.166 (0.013, 0.319)	0.034	–1.774 (–1.945, –1.602)	0.001
Year of diagnosis ≥2014	–0.141 (-0.271, -0.010)	0.035	–1.352 (–1.502, –1.203)	0.001
Hepatitis B	**-**	**-**	0.286 (0.113, 0.459)	0.001
Hepatitis C	0.206 (0.051, 0.361)	0.009	1.486 (1.288, 1.683)	0.001
ALBI grade				
1	Ref.		Ref.	
2	0.212 (0.075, 0.350)	0.003	1.735 (1.579, 1.892)	0.001
3	0.326 (0.069, 0.584)	0.013	2.576 (2.227, 2.876)	0.001
Tumour size (per cm)	0.191 (0.141, 0.242)	0.001	1.184 (1.124, 1.244)	0.001
Tumour number				
Single	Ref.		Ref.	
2 or 3 nodules	0.301 (0.149, 0.454)	0.001	1.729 (1.550, 1.907)	0.001
4+ nodules	0.812 (0.514, 1.110)	0.001	3.846 (3.449, 4.244)	0.001
Constant	–0.306 (-0.541, –0.072)		54.47 (53.93, 55.00)	

Coefficients refer to the last step of backward selection.

To obtain the cure probability for a specific clinical condition, coefficients must be multiplied by the value of the variable + the constant value. The cure proportion is then obtained by the following equation: exp(–exp(constant + *x*_1_*b*_1_ + *x*_2_*b*_2_ + … *x*_n_*b*_n_)). Ref = 0.

YLLs are approximated using coefficients from the GLM through the following equation: constant + *x*_1_*b*_1_ + *x*_2_*b*_2_ + … *x*_*n*_*b*_*n*.._ Ref = 0.

The 26% of cure threshold (equal to the 75th percentile) was arbitrarily set to individualise ideal clinical situations determining cure (Table 4). Notably, patients with very-early disease and ALBI graded 1 had the highest likelihood of achieving cure, up to 39.8% in the most recent era (since 2014 onwards). Additionally, in female patients and/or in absence of hepatitis C infection, cure probabilities >26% were observed also for ALBI graded as 2 and in the presence of mild enlargement of tumour burden.

**Table 4:** Clinical situations where the cure probability was >26% (75th percentile).

Clinical features				Cure prob. (95% C.I.)	Years of life lost (95% C.I.)		
Gender	Hepatitis C	Tumour burden	ALBI	All patients	Age < 60 years	Age 60–70 years	Age > 70 years
Female	Negative	Single <2 cm	1	39.8% (31.3, 48.3)	19.5 (18.0, 21.1)	13.0 (11.4, 14.5)	6.5 (5.0, 8.0)
Female	Negative	Single 2–3 cm	1	35.7% (27.2, 44.3)	20.3 (18.7, 21.8)	13.7 (12.2, 15.2)	7.2 (5.7, 8.7)
Male	Negative	Single <2 cm	1	33.8% (25.3, 42.4)	17.8 (16.2, 19.3)	11.2 (9.7, 12.8)	4.7 (3.2, 6.2)
Female	Positive	Single <2 cm	1	32.3% (24.0, 40.9)	21.0 (19.5, 22.6)	14.5 (13.0, 16.0)	8.0 (6.4, 9.5)
Female	Negative	Single <2 cm	2	32.1% (23.8, 40.7)	21.3 (19.7, 22.8)	14.8 (13.2, 16.3)	8.2 (6.7, 9.7)
Male	Negative	Single 2–3 cm	1	29.6% (21.5, 38.2)	18.5 (17.0, 20.0)	12.0 (10.4, 13.5)	5.4 (3.9, 6.9)
Female	Negative	2-3 nodules <2 cm	1	28.9% (20.8, 37.4)	21.3 (19.7, 22.8)	14.7 (13.2, 16.3)	8.3 (6.7, 9.7)
Female	Positive	Single 2–3 cm	1	28.1% (20.2, 36.7)	21.7 (20.2, 23.3)	15.2 (13.7, 16.7)	8.7 (7.1, 10.2)
Male	Positive	Single <2 cm	1	26.3% (18.5, 34.8)	19.3 (17.7, 20.8)	12.7 (11.2, 14.2)	6.2 (4.7, 7.7)

Estimates consider that clinical features not included were at their mean and that patients were treated from 2014 onward.

### Comprehensive evaluation of YLL

Estimates on YLL derived from the flexible parametric cure model are depicted in Fig. 1b. The 25th percentile corresponded to 11.5 YLLs and the 75th percentile to a value of 22.7 YLLs. These values were re-fitted through the generalised linear model reported in Table 3 obtaining an *R*^2^ of 0.966 (Supplementary Figure). Setting a threshold for YLL to 11.5 years (equal to the 25th percentile), the number of YLL remained under this value mainly in patients aged >70 years.

A simple calculator that permits estimation ‘chance of cure’ for all potentially curative therapies is available at https://prediction-models.liverpool.ac.uk/curative.

## Discussion

A comprehensive evaluation of cancer treatment success should rely not only on crude survival estimates, but should also consider the characteristics of the treated population. Faced with the same clinical picture, different aged patients may have the same survival in absolute terms but have a considerable difference in terms of average life-expectancy. The quantification of clinical success through cure estimation and quantification of lifespan after HCC diagnosis represents an advance on our understanding of the impact of this cancer on patient expectations.

In the treatment of HCC, ablative therapies represent the standard of care for patients at an early stage, either as an alternative to surgery or as a bridge to liver transplantation. In clinical practice, ablated patients are often older, with an impaired liver function and a tumour location, which prevents liver transplantation or hepatic resection [11]. The present study suggests that the cure rate for HCC treated with RFA or MWA is in the order of 20% rising to nearly 40% among those with the smallest tumours and best liver function—that is ‘ideal candidates’ for ablation. An overall survival at a median follow-up of nearly 4 years of 50% suggests that our population is typical of current practice and that the estimated cured proportions are those attainable in most potential candidates, ideal or not, for ablation. Thus, while the figures for cure rate are encouraging they should be taken in conjunction with the corresponding figures for years of life lost consequent to HCC development. In the present series, the YLL are around 17 rising to 20 in younger patients with the largest tumours and worst liver function. These disappointing results underline that, out of the guidelines recommendations, ablation has a very low probability of cure. All these figures deserve some comparison with the other therapies for HCC.

For more than a decade there has been a debate as to whether ablation or resection are optimal for various patient groups. For those with very-early stage disease ablation is clearly superior in terms of cost-effectiveness. However, relatively few patients have characteristics allowing consideration of one or the other treatment. The current cure estimation of 18.3% seems comparable with previous estimations of cure after resection (about 17%), suggesting that the common claim that both approaches are ‘potentially curative’ should be treated with caution [4]. In fact, the figures for cure rate after resection were based on a cohort aged 60 years (81.8% males), mostly HBV (55.3%) in ALBI grade of 1 (65.8%), with single nodule (77.4%) and a median tumour size of 4.0 cm. Including these features in the present model returns a cure estimation of 11.3% after ablation with an estimated 19.6 YLLs. Conversely, applying the present case-mix characteristics to previous modelling after resection, the large majority of patients would belong to the ERASL low-risk class of recurrence, returning a cure probability of 25.5%. Thus, cure fraction estimations provide novel insights into the eventual superiority of one of these treatments for the individual patient [12]. It becomes more important to be aware of the possibilities of being cured with one of the treatments rather than focussing on generalisations as to the superiority of resection or ablation. From this point of view, it is noticeable that after ablation it takes 10 years before HCC cure can be claimed with 90% certainty, whereas after resection it takes about 7.5 years [4]. This difference can be the consequence of the different degree of the underlying liver dysfunction (ALBI 1 grade 65.8% in resected patients and 37.7% in ablated patients), which disadvantages ablated patients who obtain statistical cure with more difficulty, finally prolonging the time-to-cure. In both cases, the fact that the time-to-cure was longer than the median follow-up suggests that surveillance for recurrence after surgery or ablation must be prolonged, as neither of the treatments cure the underlying disease that produced the tumour. In this sense, transplant is the solution [3].

Strengths of the study include the contemporary composition of the patient set, the broad international base (66% Eastern, 44% Western) and the wide variety of aetiologies suggesting that the results are relevant to current clinical practice in the global setting. It should be noted that providing survival and the diagnosis of relapse are accrued prospectively the otherwise retrospective nature of our study is a necessary requirement for this type of analysis and not a limitation. Nonetheless, we are aware that all prognostic models will require periodic review to confirm their applicability to contemporary practice. In the present study the calibration of the implied model for ‘years of life lost’ was excellent but we will need to keep this under review as liver function, consequent upon anti-viral therapy, improves and technical advances are made in ablative technologies. The ALBI score, a modern refinement of the CP score [8], was employed for assessment of liver function because it has been extensively validated and finds particular use in early HCC because it can provide prognostic discrimination in patients who, by CPS have ‘normal’ liver function (‘CP-5’) [8, 13, 14]. Of course, operator experience and available facilities may influence outcome but all our centres have extensive experience of RFA and most authorities would agree patients with early HCC should not be treated outside such centres.

Some limitations should be acknowledged. Although based on a very large sample size, the present study is not population-based. This limitation does however permit us to use more detailed and verified data for the analyses. Despite this, additional data on proximity to vessels, a known treatment response factor, would probably have further refined cure and YLL estimates. A second limitation is that cure probability and lifespan calculations are based on the projection of survival curves, which represent the estimated rather than observed results. To acquire the latter would require an extremely long follow-up such that the results would be out-of-date at the time of the assessment. The third limitation is that the YLL estimate is affected not only by the survival of the HCC cohort but also by the life-expectancy of the reference cohort. Life-expectancy of the reference population is influenced by the cancer itself, so that cure probabilities and YLL may have been underestimated [15]. However, a single cancer has a relatively small effect on the life tables of the general population, which include all causes of death, so that it is likely that this unavoidable bias was minimal. Our study is also limited by absence of data in patients with the important aetiology of NAFLD. Caution should be exercised in extrapolating our results in patients with alcoholic liver disease or NAFLD and we acknowledge that the models might need revision over time. Finally, and most importantly, the general population was considered here as the reference population whereas a population of subjects suffering from chronic hepatitis or cirrhosis would best serve for the purpose [4]. However, to the best of our knowledge, no population-based statistics stratified by chronic liver disease are available to support such a requirement. We acknowledge that a fully matched reference population stratified by aetiology as well as fibrosis stage will probably provide a more accurate picture of cure probabilities after ablation for HCC.

Our results confirm that LT holds first place hierarchically. The reported dramatic improvement (up to 75%) [3] highlights that LT is the real curative treatment for HCC, because pre-existing, but not clinically detectable, metastatic disease is removed and liver function is improved. Determinants of cure and of YLL after ablation were the same as the known risk-factors for crude survival estimates, with the adjunction of age and gender. Consequently, hepatitis status, tumour features and liver function provided different survivals, which returned different cure estimates and YLL when age and gender were considered to measure relative survivals. Similar results for patients treated with surgical resection have already been provided [4]. It is of note that hepatitis C was an adverse factor in the multivariable model so that it is likely that cure (and resection) rate will improve with the complete eradication of HCV infection with DAAs over the next few years [16]. Additionally, we observed an increased probability of being cured in the most recent years (namely, from 2014 onwards). This is consistent with results observed for resections performed in the modern anti-viral eras for both HBV and HCV, and are consistent with previous observations coming from a large National database [17]. These are probably related to improvements in technique and the management of the underlying liver disease [18].

In conclusion, here we provided the estimation of being cured from HCC with percutaneous ablation. Results highlight that within guidelines’ recommendations, the probability of being cured can be up to 40%, but that it can take up to 10 years to obtain at least 90% of certainty. The amount of YLL would be minimal in patients aged above 70 years. The combination of the present model with that after liver resection and liver transplantation, available at https://prediction-models.liverpool.ac.uk/curative, provides a comprehensive model of cure after treatments considered ‘potentially curative’.

## Supplementary information

Supplementary methods

Supplementary figure 1

## Data Availability

All reasonable requests will be considered on application to the corresponding author and subject to review by the relevant Ethics Committee.
