# Supplementary material for: ‘Potentially curative therapies’ for hepatocellular carcinoma: how many patients can actually be cured?
Source: Br J Cancer. 2023 Feb 17;128(9):1665–71. doi: 10.1038/s41416-023-02188-z (PMC10133312; doi:10.1038/s41416-023-02188-z)
Supplement: Supplementary file 1 — Supplementary methods [file 41416_2023_2188_MOESM1_ESM.docx]

**‘Potentially curative therapies’ for hepatocellular carcinoma: how many patients can actually be cured?**

Alessandro Cucchetti, Omar Elshaarawy, Guohong Han, Charing CN Chong, Carla Serra, Joanne Marie O’Rourke, Richard Crew, Cristina Felicani, Giorgio Ercolani, Tahir Shah, Arndt Vogel, Paul BS Lai, Philip J Johnson

***Supplementary Material:***

***Methods:***

To assess the curative ability of ablation we accessed data from 1571 HCC patients treated in 5 centres (2004-2018) receiving radiofrequency (RFA) or microwave (MWA) ablation were used to fit a flexible parametric survival model to estimate relative survival, cure fraction and years of life lost (YLL) such that the chance of cure and years of life lost could be estimated by reference to healthy populations, based on national life-tables. Patients with macro-vascular invasion, extra-hepatic disease or Child–Pugh class >8 were excluded. The results of this analysis were then combined with our previous estimations of cure fraction in liver transplantation and resection to provide a simple calculator applicable to all patients underpoing potentially curative therapies

The aim of the study was to develop a statistical model that permits estimation of the ‘chance of cure’ following ablation, the third ‘potentially curative’ approach. This would complement our previous work so as to provide clinicians a ‘calculator’ to readily assess the impact of any of the various PCTs ‘on the chances of cure’ based on routinely available clinical features.

Our results also confirm that LT holds first place hierarchically. The reported dramatic improvement (up to 75%)(3) highlights the fact that LT is the real curative treatment for HCC, because pre-existing, but not clinically detectable, metastatic disease is removed and liver function is improved. From these observations a coherent and quantitative summary of the current status of treatment of early HCC can be drawn. Ablation (and resection) offer a 20% chance of cure, the figure being limited by pre-existing liver disease and residual micro-metastatic disease. The former is most likely improving over time so that chance of cure will likely improve in parallel.

**Supplementary Figure Legend:** Calibration between YLLs resulting from the parametric survival curve and that estimated from generalized linear model necessary to provide a reproducible model.
