# Supplementary figures and images for: ‘Potentially curative therapies’ for hepatocellular carcinoma: how many patients can actually be cured?
Source: Br J Cancer. 2023 Feb 17;128(9):1665–71. doi: 10.1038/s41416-023-02188-z (PMC10133312; doi:10.1038/s41416-023-02188-z)

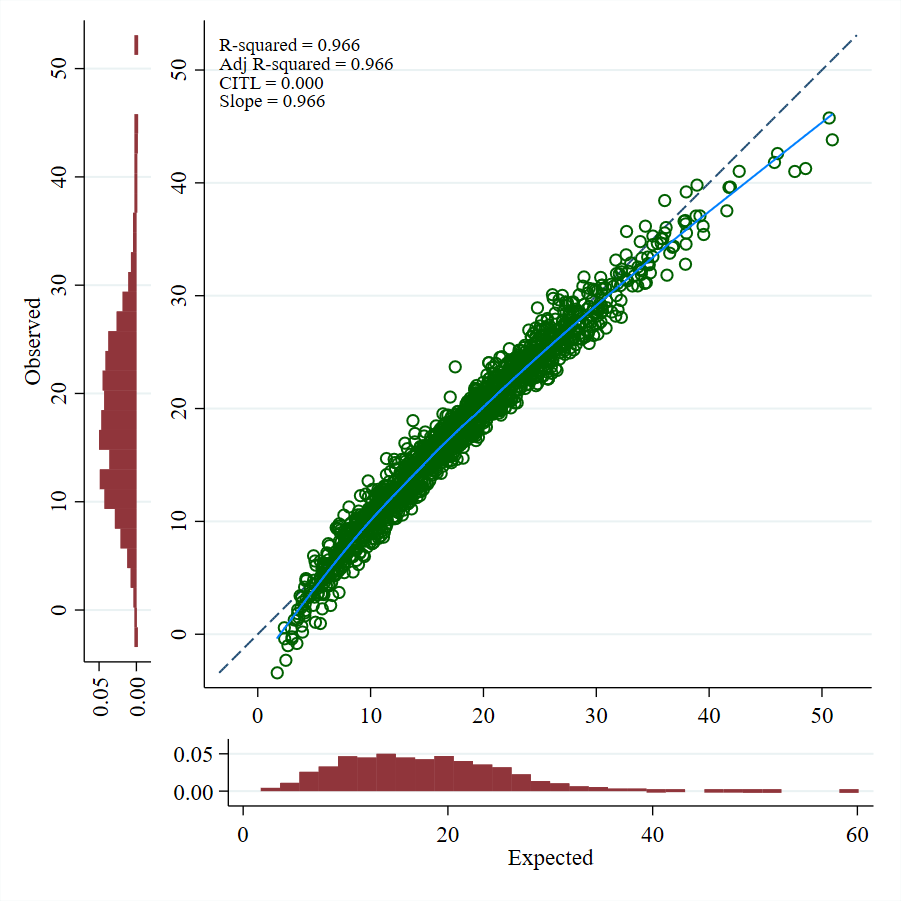

Supplement: Supplementary file 2 — Supplementary figure 1 [file 41416_2023_2188_MOESM2_ESM.png]
